# Supplementary material for: Generalizable clinical note section identification with large language models
Source: JAMIA Open. 2024 Aug 13;7(3):ooae075. doi: 10.1093/jamiaopen/ooae075 (PMC11319784; doi:10.1093/jamiaopen/ooae075)
Supplement: ooae075_Supplementary_Data [file ooae075_supplementary_data.docx]

**Appendices**

**1. Discharge dataset section mappings, definitions, and examples, with sensitive information censored/modified, e.g., [ID], [DATE], [TIME], and sections shortened for readability.**

| Original section type | Mapped section type | Description | Example |
| --- | --- | --- | --- |
| Unknown | Unknown | the given information is insufficient for inferring the section's section name | [ID]  PRGH  [DATE] [TIME]  CORONARY ARTERY DISEASE  Unsigned  DIS |
| Subsection | Unknown | the given information is insufficient for inferring the section's section name | COMPLICATIONS :  1. Post operative bleeding requiring return to the Operating Room for mediastinal exploration and evacuation of hematoma .  2. Profound leukopenia thought secondary to Diuril administration . |
| Admit Date | Admit date | the date of the patient's admit, example section headings include 'admission date' | Admission Date :  [DATE]  Report Status : |
| Discharge Date | Discharge date | the date of the patient's discharge, example section headings include 'discharge date' | DISCHARGE PATIENT ON :  [DATE] AT [TIME]  CONTINGENT UPON HO evaluation  WILL D/C ORDER BE USED AS THE D/C SUMMARY :  YES  CODE STATUS :  Full code |
| Reason for Admission | Reason for admission | the reason why the patient was admitted to the hospital, example section headings include 'chief complaint', 'history of present illness', 'reason for hospitalization' | Patient States Complaint :  SYNCOPAL EPISODE |
| Discharge Instructions | Discharge instructions | guidelines and information provided to a patient when they are being discharged from the hospital, example section headings include 'todo', 'plan', 'diet', 'discharge instructions' | DIET :  House / ADA 2100 cals / dy  RETURN TO WORK :  Immediately |
| Discharge Diagnoses | Discharge diagnosis | diagnosis assigned to a patient at the time of their discharge from the hospital, example section headings include 'principle discharge diagnosis', 'discharge diagnosis' | Diagnosis :  Epigastric discomfort |
| Discharge Medications | Discharge medications | medications prescribed to a patient at the time of their discharge from the hospital, example section headings include 'discharge medications', 'medications on discharge' | DISCHARGE MEDICATIONS :  ALBUTEROL INHALER 2 PUFF INH QID |
| Admission Diagnoses | Admission diagnosis | medical condition or diagnosis that leads to a patient's admission to a healthcare facility, example section headings include 'admit diagnosis', 'principle diagnosis' | ADMIT DIAGNOSIS :  atypical cp |
| Procedures | Patient procedures | medical interventions, treatments, or actions performed by healthcare professionals to diagnose, treat, manage, or prevent various medical conditions, example section headings include 'operations and procedures', 'procedures', 'principle procedures', 'major surgical or invasive procedures', 'special procedures and instructions' | PRINCIPLE PROCEDURE :  Right total hip replacement . |
| Hospital Course | Hospital course | the sequence of events and medical interventions that occur during a patient's stay in a hospital, example section headings include 'hospital course', 'brief resume of hospital course' | HOSPITAL COURSE :  Patient underwent a myomectomy on [DATE] .  Operative findings included a 10 cm fundal fibroid which was adhered to the cul-de-sac .  There were normal ovaries and tubes otherwise . |
| Past Medical History | Past medical history | a patient's documented medical history of illnesses, conditions, surgeries, and significant medical events that have occurred prior to the current medical encounter, example section headings include 'past medical history' | PAST MEDICAL HISTORY :  Significant for manic depressive illness in the past . |
| Past Surgical History | Past surgical history | a patient's documented history of surgical procedures and operations they have undergone in the past, example section headings include 'past surgical history' | PAST SURGICAL HISTORY :  Status post cesarean section . |
| History | History of present illness | the patient's history of present illness, example section headings include 'history of present illness' | HISTORY OF PRESENT ILLNESS :  This is an [AGE] year old man initially admitted to the Plastic Surgery Service for evaluation of a left facial mass .  Subsequently , CMED CCU was consulted and he was transferred to our Service postoperatively . |
| Physical | Physical examination | the patient's physical examination, example section headings include 'physical examination', 'review of systems' | PHYSICAL EXAMINATION :  She is afebrile and vital signs were stable .  She has some history of hearing loss . |
| Social History | Social history | a patient's background and lifestyle factors that can have a significant impact on their health and well-being, example section headings include 'social history' | SOCIAL HISTORY :  Stopped smoking many years ago . |
| Family History | Family history | a record of health-related information about an individual's immediate family members, such as parents, siblings, and children, example section headings include 'family history' | FAMILY HISTORY :  Noncontributory . |
| Allergies | Allergies | a dedicated portion of a patient's medical record or health profile that contains information about their known allergies, example section headings include 'allergies' | ALLERGIES :  No known drug allergies . |
| Followup | Follow-up | the follow-up information given to the patient after discharge, example section headings include 'follow up', 'follow up appointment', 'follow up services', 'instructions', 'dispositions', 'plan on discharge', 'patient dispositions' | FOLLOW UP APPOINTMENT ( S ) :  Dr. [NAME] 2 weeks , No Known Allergies |
| Disposition | Follow-up | the follow-up information given to the patient after discharge, example section headings include 'follow up', 'follow up appointment', 'follow up services', 'instructions', 'dispositions', 'plan on discharge', 'patient dispositions' | DISPOSITION :  Home |
| Medications | Admission medications | substances, typically in the form of drugs or pharmaceuticals, that are used to diagnose, treat, manage, or prevent medical conditions, example section headings include 'medications', 'medications on admission' | MEDICATIONS ON ADMISSION :  Maxzide , Lisinopril , Cardia , Aspirin , Citracal , Fosamax , and Levoxyl . |
| Gynecologic History | Gynecologic history | a medical record or health profile pertains to a patient's reproductive and gynecological health | PAST OB / GYN HISTORY :  Normal Paps , mammograms normal . |
| Other Diagnoses | Other diagnosis | diagnoses that are not discharge diagnosis or admission diagnosis, example section headings include 'associated diagnosis', 'other diagnosis', 'secondary diagnosis' | OTHER PROBLEMS :  None . |
| Service | Patient service | services done for the patient, example section headings include 'service' | Service :  ONC |
| Condition | Discharge condition | patient's condition on discharge, example section headings include 'discharge condition', 'condition on discharge' | DISCHARGE CONDITION :  Stable |
| Studies | Lab studies | laboratory studies for the patient, example section headings include 'laboratory data', 'laboratory studies', 'pertinent results' | LABORATORY DATA :  Sodium [NUMBER] , potassium [NUMBER] , chloride [NUMBER] , bicarb [NUMBER] , BUN [NUMBER] , creatinine [NUMBER] , and glucose [NUMBER] .  The UA was negative . |
| Comments | Patient comments | comments left for patients, example section headings include 'additional comments', 'summary', 'addendum' | ADDITIONAL COMMENTS :  If you have more shortness of breath , please contact your primary care physician / oncologist .  If you have chest pain , diaphoresis , nausea / vomiting , please return to emergency room .  No changes were made to your medication regimen . |
| Admit Physician | Admitting physician | the physician who admits the patient | Accepting / DH Admitting Physician :  [NAME]  PCP Name :  [NAME]  Provider Number :  [ID]  This report was created by [NAME] [DATE] [TIME] |
| Attending | Attending physician | the patient's attending physician, example section headings include 'attending' | Attending :  [NAME] , M.D.  CODE STATUS :  Full code |

**2. Progress dataset section mappings, definitions, and examples, with sensitive information censored/modified, e.g., [ID], [DATE], [TIME], and sections shortened for readability**

| Original section type | Mapped section type | Description | Example |
| --- | --- | --- | --- |
| Patient Surgical History | Patient surgical history | a patient's documented history of surgical procedures and operations they have undergone in the past, example section headings include 'PSH', 'PShx', 'SH' | Chief Complaint: pancreatitis, alcohol withdrawal |
| Assessment and Plan | Assessment and plan | where healthcare providers summarize their assessment of the patient's medical condition and outline the plan for diagnosis, treatment, and management, example section headings include 'assessment and plan' | Assessment and Plan  DEEP VENOUS THROMBOSIS (DVT), UPPER EXTREMITY  .H/O CANCER (MALIGNANT NEOPLASM), COLORECTAL (COLON CANCER) |
| Physical Exam | Physical examination | the patient's physical examination, example section headings include 'physical examination' | Physical Examination  GEN: Intubated, in no distress, opens eyes to command  SKIN:No rashes, no cyanosis, no ulcerations  CHEST: ronchi R |
| Radiology | Radiology | where information related to radiological studies and imaging procedures is recorded, example section headings include 'CXR', 'radiology' | Imaging:  [DATE]  Right Knee film:  IMPRESSION: Unremarkable study. No evidence of acute traumatic injury |
| Laboratory | Laboratory | laboratory test results for the patient, example section headings include 'WBC', 'labs', 'ABG' | Labs  [NUMBER] K/uL  [NUMBER] g/dL  [NUMBER] mg/dL  [NUMBER] mg/dL  [NUMBER] Eq/L  [NUMBER] mEq/L  [NUMBER] mg/dL  [NUMBER] mEq/L |
| Vital Sign | Vital sign | it documents various physiological measurements that provide important information about a patient's overall health and stability, example section headings include 'vital signs' | Flowsheet Data as of [DATE] [TIME]  Vital signs  Hemodynamic monitoring  Fluid balance  Tmax: [NUMBER]  C ([NUMBER]  Tcurrent: [NUMBER]  C ([NUMBER]  HR: [NUMBER] ([NUMBER] - [NUMBER]) bpm |
| Medications | Medications | substances, typically in the form of drugs or pharmaceuticals, that are used to diagnose, treat, manage, or prevent medical conditions, example section headings include 'last dose of antibiotics', 'current medications' | Last dose of Antibiotics:  Bactrim (SMX/TMP) - [DATE] [TIME]  Infusions:  Other ICU medications:  Metoprolol - [DATE] [TIME]  Other medications: |
| Review of System | Review of system | a systematic inquiry or checklist used by healthcare providers, typically during a medical history-taking process, to gather information about a patient's overall health and any potential symptoms or issues they may be experiencing, example section headings include 'Review of systems is unchanged from admission except as noted below Review of systems' | Review of systems is unchanged from admission except as noted below  Review of systems: |
| Past Medical History | Past medical history | a patient's documented medical history of illnesses, conditions, surgeries, and significant medical events that have occurred prior to the current medical encounter, example section headings include 'changes to medical', 'PMHx', 'medical' | PMHx:  PMH: CAD s/p MI in [DATE] (s/p stents), h/o PE in '[NUMBER], HTN, DM,  cecal adenoma |
| Chief Complaint | Chief complaint | the reason why the patient was admitted to the hospital, example section headings include 'chief complaint' | Chief Complaint: chest pain, SOB |
| History of Present Illness | History of present illness/24 hour events | the patient's history of present illness, or the patient events that happened in the last 24 hours, example section headings include 'history of present illness', 'HPI', '24 hour events' | 24 Hour Events:  Following sheath pull patient mom [NAME] lost consciousness which she  regained within seconds after receiving one dose of atropine. |
| Allergies | Allergies | a dedicated portion of a patient's medical record or health profile that contains information about their known allergies, example section headings include 'allergies' | Allergies:  Penicillin V  Unknown; |
| Family History | Family history | a record of health-related information about an individual's immediate family members, such as parents, siblings, and children, example section headings include 'family history' | family history: none |
| Social History | Social history | a patient's background and lifestyle factors that can have a significant impact on their health and well-being, example section headings include 'Soc', 'SH', 'PSH' | SH: none |
| Addendum | Unknown | the given information is insufficient for inferring the section's section name | Comments:  Communication: Comments:  Code status: Full code  Disposition:ICU |
| Other | Unknown | the given information is insufficient for inferring the section's section name | [image002.jpg]  [DATE] [TIME]  [DATE] [TIME] |
| Unknown | Unknown | the given information is insufficient for inferring the section's section name | Code: Full (discussed with son) |

**3. Full prompt example**

You are a helpful assistant. You are an experienced clinician and you are familiar with writing and understanding clinical notes.

A clinical note contains multiple sections like family history, allergies and history of present illness.

Given a clinical note as an input, please separate the note into sections and output their section names. Also specify where the section starts and ends. Use section names from one of the following:

Attending physician: the patient's attending physician, example section headings include 'attending'

Unknown: the given information is insufficient for inferring the section's section name Admit date: the date of the patient's admit, example section headings include 'admission date'

Discharge date: the date of the patient's discharge, example section headings include 'discharge date'

Reason for admission: the reason why the patient was admitted to the hospital, example section headings include 'chief complaint', 'history of present illness', 'reason for hospitalization'

Discharge instructions: guidelines and information provided to a patient when they are being discharged from the hospital, example section headings include 'todo', 'plan', 'diet', 'discharge instructions'

Discharge diagnosis: diagnosis assigned to a patient at the time of their discharge from the hospital, example section headings include 'principle discharge diagnosis', 'discharge diagnosis'

Discharge medications: medications prescribed to a patient at the time of their discharge from the hospital, example section headings include 'discharge medications', 'medications on discharge'

Admission diagnosis: medical condition or diagnosis that leads to a patient's admission to a healthcare facility, example section headings include 'admit diagnosis', 'principle diagnosis'

Patient procedures: medical interventions, treatments, or actions performed by healthcare professionals to diagnose, treat, manage, or prevent various medical conditions, example section headings include 'operations and procedures', 'procedures', 'principle procedures', 'major surgical or invasive procedures', 'special procedures and instructions'

Hospital course: the sequence of events and medical interventions that occur during a patient's stay in a hospital, example section headings include 'hospital course', 'brief resume of hospital course'

Past medical history: a patient's documented medical history of illnesses, conditions, surgeries, and significant medical events that have occurred prior to the current medical encounter, example section headings include 'past medical history'

Past surgical history: a patient's documented history of surgical procedures and operations they have undergone in the past, example section headings include 'past surgical history'

History of present illness: the patient's history of present illness, example section headings include 'history of present illness'

Physical examination: the patient's physical examination, example section headings include 'physical examination', 'review of systems'

Social history: a patient's background and lifestyle factors that can have a significant impact on their health and well-being, example section headings include 'social history'

Family history: a record of health-related information about an individual's immediate family members, such as parents, siblings, and children, example section headings include 'family history'

Allergies: a dedicated portion of a patient's medical record or health profile that contains information about their known allergies, example section headings include 'allergies'

Follow-up: the follow-up information given to the patient after discharge, example section headings include 'follow up', 'follow up appointment', 'follow up services', 'instructions', 'dispositions', 'plan on discharge', 'patient dispositions'

Admission medications: substances, typically in the form of drugs or pharmaceuticals, that are used to diagnose, treat, manage, or prevent medical conditions, example section headings include 'medications', 'medications on admission'

Gynecologic history: a medical record or health profile pertains to a patient's reproductive and gynecological health

Other diagnosis: diagnoses that are not discharge diagnosis or admission diagnosis, example section headings include 'associated diagnosis', 'other diagnosis', 'secondary diagnosis' Patient service: services done for the patient, example section headings include 'service'

Discharge condition: patient's condition on discharge, example section headings include 'discharge condition', 'condition on discharge'

Lab studies: laboratory studies for the patient, example section headings include 'laboratory data', 'laboratory studies', 'pertinent results'

Patient comments: comments left for patients, example section headings include 'additional comments', 'summary', 'addendum'

Admitting physician: the physician who admits the patient

Attending physician: the patient's attending physician, example section headings include 'attending'

For example, the output format should be

Section 1: "Social history"

Starts at: "SOCIAL HISTORY:"

Ends at: "no alcohol."

Section 2: "Family history"

Starts at: "FAMILY HISTORY:"

Ends at: "parents do not have diabetes."

Input:

[clinical note]

Output:

**4. Example output (made up)**

Section 1: "Admit date"

Starts at: "Admission Date:"

Ends at: "2001-01-01"

Section 2: "Social history"

Starts at: "SOCIAL HISTORY:"

Ends at: "alcohol"

Section 3: "History of present illness"

Starts at: "HISTORY OF PRESENT ILLNESS:"

Ends at: "knee replacement surgery."

Section 4: "Family history"

Starts at: "FAMILY HISTORY:"

Ends at: "none."
